# Facile Solid-State Chemical Synthesis of CoMoO_4_ Nanorods for High-Performance Supercapacitors

**DOI:** 10.3390/molecules29061369

**Published:** 2024-03-19

**Authors:** Rui Yu, Xiaoyan Lu, Zhenjiang Lu, Yali Cao

**Affiliations:** State Key Laboratory of Chemistry and Utilization of Carbon Based Energy Resources, College of Chemistry, Xinjiang University, Urumqi 830046, China13609989486@163.com (X.L.)

**Keywords:** cobalt molybdate, supercapacitor, electrode material, solid-phase chemical reaction

## Abstract

The development of electrode materials with excellent performance serves as the key for researchers to enhance the energy density of supercapacitors. Cobalt molybdate (CoMoO_4_) nanomaterials have been regarded as one of the most prospective electrode materials for supercapacitors due to their high theoretical capacitance and excellent electrical conductivity. In this paper, three kinds of CoMoO_4_ nanorods were prepared directly via simple and environmentally friendly solid-phase chemical reactions with solid inorganic salts as raw materials. According to X-ray powder diffraction (XRD) and scanning electron microscopy (SEM) test results, different reagents had certain effects on the size and morphology of CoMoO_4_, and these affected its electrochemical performance. In particular, the samples prepared with Co(NO_3_)_2_·6H_2_O as raw material took on a more uniform micromorphology, with a better crystallinity. Simultaneously, electrochemical test results showed that the samples synthesized with Co(NO_3_)_2_·6H_2_O presented relatively good electrical conductivity and a large specific capacitance (177 F g^−1^). This may be due to the nitrates reacting more slowly during the reaction and the crystals having difficulty aggregating during growth. Therefore, the structure of the prepared CoMoO_4_ nanomaterial was more uniform, and it was resistant to collapse during the charging and discharging process; thus, the capacitor presents the best performance.

## 1. Introduction

With the rapid advancement in the global economy and the continuously deteriorating environment, as well as diminishing non-renewable energy sources such as coal, natural gas, and petroleum, both energy and environmental issues have become two critical challenges faced by human society concerning our sustainability [1,2,3]. Due to their geographical limitations and intermittent nature, wind energy, solar energy, and other green energy sources often need to be converted into electricity for storage via energy storage equipment. Currently, electrochemical energy storage technology is considered one of the most effective energy storage methods [4,5]. However, low power density, poor safety, and other reasons have severely restricted the further commercialized application of secondary batteries. Moreover, secondary batteries mainly store electric energy via charge transfer, and their service life and stability are severely subject to temperature and other external environments. As a result, there is an urgent need for researchers to develop an energy storage device with a long service life and high reliability that can adapt to a high-power operation environment [6,7,8]. A supercapacitor, also known as an electrochemical capacitor, serves as a new kind of energy storage device between the traditional capacitor and battery. Its unique solid–liquid interface ion adsorption energy storage mechanism has broken through the limitations of traditional battery-type devices through active phase-change material energy storage. In this way, it presents larger power density, faster charge and discharge rates, broader utilization temperature range, longer cycle life, and greater safety measures, thereby gaining widespread attention from scientific researchers [9,10,11].

The key to progress for supercapacitors lies in the design and development of electrode materials with excellent performance and a low price [12,13,14]. At present, the electrode materials of supercapacitors are mainly carbon-based materials, conductive polymers, and transition metal oxides. Among them, carbon-based materials, mainly including activated carbon, carbon nanotubes, graphene, carbon matrix composites, etc., with a large specific surface area and high conductivity, have been commercially produced. Nevertheless, activated carbon has limited capacity in the aspect of capacitive storage due to its energy storage mode, and the failure to achieve high energy storage and other issues have seriously restricted its commercialization. Carbon nanotubes, graphene, and carbon matrix composites still cannot meet the requirements for commercialized manufacture due to their complex preparation process and high preparation costs [15,16,17]. Conductive polymers can exhibit a relatively high energy density by storing energy through pseudocapacitance behavior. However, the capacitive storage mechanism of conductive polymer electrode materials occurs through the continuous embedding and removal of ions; this process damages the structure of the conductive polymer, thus worsening its stability. Simultaneously, conductive polymers present a small specific surface area, and their synthesis process involves toxic agents, such as aniline, pyridine, and pyrrole, which are not conducive to the physical health of researchers and the requirements for environmental friendliness [18,19]. Transitional metal oxide compounds present a high theoretical capacity, low costs, environmental friendliness, etc., but their low conductivity cannot achieve high capacitance performance during practical application. According to research, the conductivity and stability of transitional metal oxide compounds can be improved effectively by adjustments in their structure, morphology, and doped heteroatoms via various preparation methods [20,21,22].

Cobalt molybdate materials, a kind of two-element transitional metal oxide, facilitate reversible redox reactions due to their abundant reserves and variable valence states of cobalt and molybdenum ions, and their electrochemical performance is usually better than that of single-component oxides. Moreover, cobalt and molybdenum ions display rapid electron transfer and good electrical conductivity due to their abilities to form empty d-orbitals after hybridization; therefore, cobalt molybdate (CoMoO_4_) has been considered one of the most prospective electrode materials for supercapacitors [23,24]. Therefore, using different methods to prepare CoMoO_4_ nanomaterials and modify them to obtain high-capacity and high-stability electrode materials has become a research hotspot in the field of supercapacitors. For example, according to Lee et al. [25], CoMoO_4_ nanorods with low crystallinity present a maximum specific capacitance of 420 F g^−1^ at a current density of 1 A g ^−1^ and have an excellent magnification capacity and cyclic stability. The CoMoO_4_ nanoclusters prepared by Chen et al. [26], through impregnation, presented good wettability, a high specific surface area, and, therefore, excellent capacity and cyclic stability. At a current density of 1 A g^−1^, the maximum specific capacitance was up to 367 F g^−1^, and the cycle efficiency was 99.8%. Jung et al. [27] introduced oxygen deficiency into an ordered three-dimensional flower-like CoMoO_4_ nanostructure, thereby effectively improving the electrical conductivity and chemical active sites of CoMoO_4_ and thus essentially improving its electrochemical performance. When the current density was 1 A g^−1^, it had a specific capacity of 531 mAh g^−1^, and the capacity retention rate was 91.03 after 10,000 cycles. However, CoMoO_4_, prepared by using the traditional liquid-phase method, served as a kind of cathode material for the capacitor and presented a poor rate capability and a low utilization rate due to its low electrode reaction kinetics. Meanwhile, CoMoO_4_ materials expanded in size during the continuous charge and discharge processes, thereby causing their structure to collapse and resulting in unsatisfactory cyclic performance. Additionally, the traditional CoMoO_4_ material preparation method features low yield, complex synthesis steps, raw material waste, high energy consumption, and other problems, and therefore cannot be applied to commercial manufacturing on a large scale [28,29].

Low-temperature solid-phase chemical synthesis is a method used to synthesize materials by the chemical reaction of solid raw materials. This method can not only quickly complete the chemical reaction at room temperature, but also can prepare nanomaterials in one step in a very short time. It has the characteristics of being convenient and fast with a simple operation, providing high yield and green environmental protection with no need to use solvents. Additionally, in the low-temperature solid-phase chemical synthesis method at near room temperature, the interaction between molecules is weakened. As a result, the process of breaking the old bond and forming the new bond is relatively mild, and the prepared products easily retain the structural characteristics of the raw material molecules, with good dispersion and wettability [30,31,32]. During recent years, nanomaterials with different morphologies, such as CoFe_2_O_4_, NiMoO_4_, and LiFePO_4_, prepared via solid-phase chemical synthesis at a low temperature have displayed excellent electrochemical properties in energy storage and transformation fields, such as lithium-ion battery, supercapacitors, and electrocatalysis [33,34,35]. Based on this, in this paper, three different CoMoO_4_ nanomaterials were synthesized by solid-phase chemical reaction using different cobalt salts, and their electrochemical properties were studied as electrode materials for supercapacitors. Structural characterization and electrochemical property tests show that the samples synthesized with Co(NO_3_)_2_·6H_2_O as a raw material presented a relatively large specific capacitance (177 F g^−1^) and stability. This may be due to the slower reaction rate of nitrates during the reaction. The structure of the prepared CoMoO_4_ nanomaterial is more uniform, so its structure is not prone to collapse during charging and discharging. At the same time, there are a large number of oxygen defects, so its capacitor performance is the best. This method can lay a theoretical foundation for the green synthesis of other two-element transitional metal oxide electrode materials and thereby provide a new train of thought.

## 2. Results and Discussion

### 2.1. Analyses of Material Composition, Structure, and Morphology

**Scheme 1:**
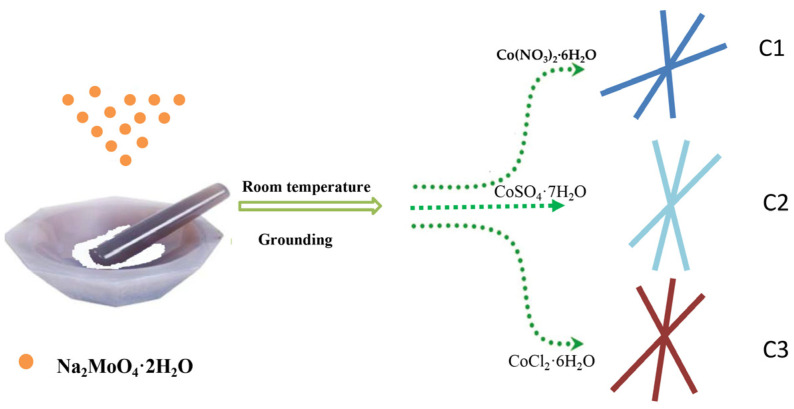
Schematic diagram showing the synthesis strategy of different cobalt molybdate nanorods.

**Figure 1:**
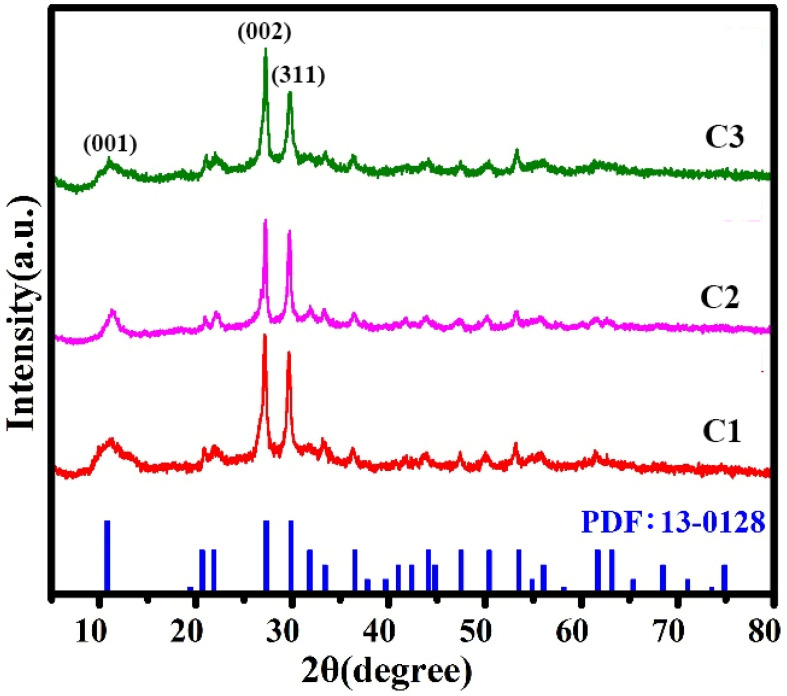
XRD patterns of CoMoO_4_ obtained by different cobalt salts: C1: Co(NO_3_)_2_·6H_2_O, C2: CoCl_2_·6H_2_O, and C3: CoSO_4_·7H_2_O.

The overall synthetic route for the different cobalt molybdate nanorods is illustrated in Scheme 1. X-ray powder diffraction (XRD) was tested to prove the phase and purity of the synthesized samples. According to Figure 1, Na_2_MoO_4_·2H_2_O reacted with Co(NO_3_)_2_·6H_2_O, CoCl_2_·6H_2_O, and CoSO_4_·7H_2_O (labeled as C1, C2, and C3, respectively) of the same substance amount; the diffraction peaks of the obtained samples were consistent with the standard card data (PDF#15−0439) concerning CoMoO_4_. The strong diffraction peaks at 13.6°, 26.6°, and 29.3° corresponded to the (001), (002), and (311) crystal faces of CoMoO_4_, respectively, indicating that samples C1, C2, and C3 prepared through solid-phase chemical reaction at ambient temperature are pure CoMoO_4_ [36]. However, the XRD peaks of three nanorods shift to different positions, which can be ascribed to the structural strain [37]. Furthermore, the diffraction peak corresponding to sample C3 was weak, thus proving that CoMoO_4_ prepared with CoSO_4_·7H_2_O as the raw material has poor crystallinity. This may be due to the high contents of water molecules and oxygen atoms in CoSO_4_·7H_2_O, which thereby affect the crystallinity of CoMoO_4_.

**Figure 2:**
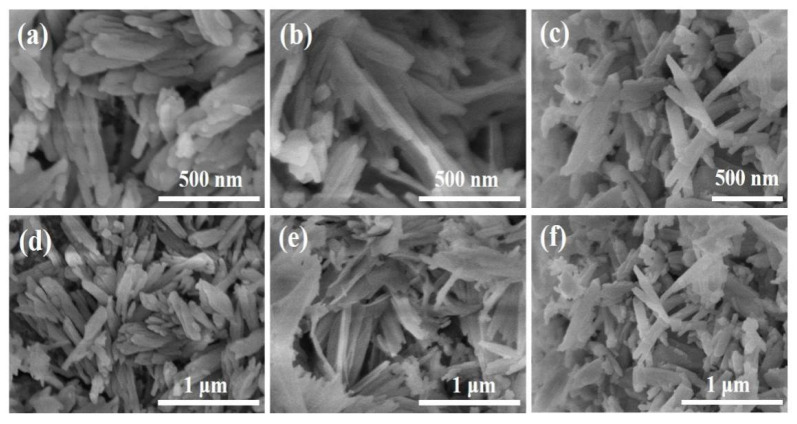
SEM graphs of (**a**,**d**) C3, (**b**,**e**) C2, and (**c**,**f**) C1.

In order to explore the influence of the raw materials for solid-phase reaction on the synthesized sample morphologies, a scanning electron microscope (SEM) analysis was conducted. According to the SEM photos in Figure 2, the samples synthesized with CoSO_4_·7H_2_O as the raw material present short rod-like structures with uneven lengths and severe agglomeration (Figure 2a,d). The preparation with CoCl2·6H2O as the raw material is uniform and has a small diameter, but there is adhesion between the rods and the dispersion is poor (Figure 2b,e). The samples synthesized from Co(NO_3_)_2_·6H_2_O present homogeneous nanorods with a diameter of about 80 nm and a length range from about 400 to 450 nm (Figure 2c,f). According to the morphologic comparison among those three samples, different raw materials have certain influences on both the size and morphology of CoMoO_4_. This may be because the rates of reactions with Na_2_MoO_4_·2H_2_O are different among the raw materials, which thereby leads to the differences in the crystal structure of CoMoO_4_. Those structure differences may influence electrochemical properties.

**Figure 3:**
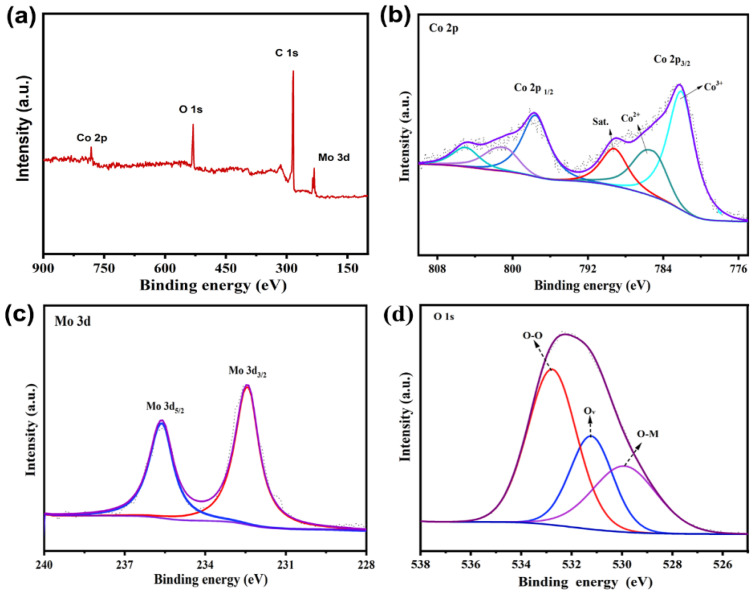
(**a**) XPS survey spectra of sample C1, (**b**–**d**) XPS spectra of Co 2p, Mo 3d, and O 1s, respectively.

To further explore the composition and valence states of the surface elements on those samples, sample C1 was tested by X-ray photoelectron spectroscopy (XPS). According to Figure 3a, there were only the characteristic peaks of Co 2p, Mo 3d, O 1s, and C1s in the survey spectrum (the C element is the introduction of a conductive adhesive during the test), further confirming that the sample contains Co, Mo, and O elements and, therefore, is pure CoMoO_4_. In order to more accurately fit the resulting energy of each element, all elements were calibrated to the C=C combined energy standard (284.8 eV). As shown in Figure 3b, in the high-resolution XPS spectra of Co 2p, the characteristic peaks at 797.7 and 782.2 eV correspond to the two spin–separation orbits of Co 2p_1/2_ and Co 2p_3/2_, respectively, with a splitting difference of 15.5 eV. It can be inferred from the fitting peaks that Co^2+^ and Co^3+^ coexist in the sample CoMoO_4_, consistent with the findings from the literature [38,39]. According to the high-resolution of Mo 3d in Figure 3c, Mo 3d_3/2_ at 232.2 eV and Mo 3d_5/2_ at 235.6 eV split through spinning into two strong characteristic peaks, and the binding energy difference between the two peaks was about 3.4 eV, corresponding to the high oxidation state characteristic peak of Mo^6+^, further proving that sample C1 is pure CoMoO_4_ [40,41]. In addition, the O 1s high-resolution spectra of sample C1 can be fitted to three characteristic peaks. In particular, the O-O characteristic peak at 532.6 eV can be attributed to chemisorbed oxygen or surface-adsorbed water. The O_v_ characteristic peak near 531.1 eV is an oxygen deficiency with low oxygen coordination (namely, oxygen vacancy), and the M-O characteristic peak near 529.3 eV is attributed to the metal–oxygen bond [42,43,44], indicating that there is an oxygen deficiency in sample C1 that facilitates in improving the specific capacity and conductivity of CoMoO_4_.

### 2.2. Electrochemical Test

**Figure 4:**
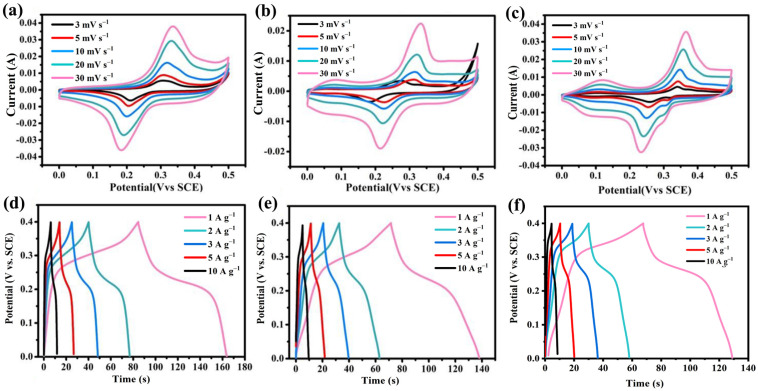
(**a**–**c**) CV curves of C1, C2, and C3 and (**d**–**f**) GCD curves of C1, C2, and C3.

**Figure 5:**
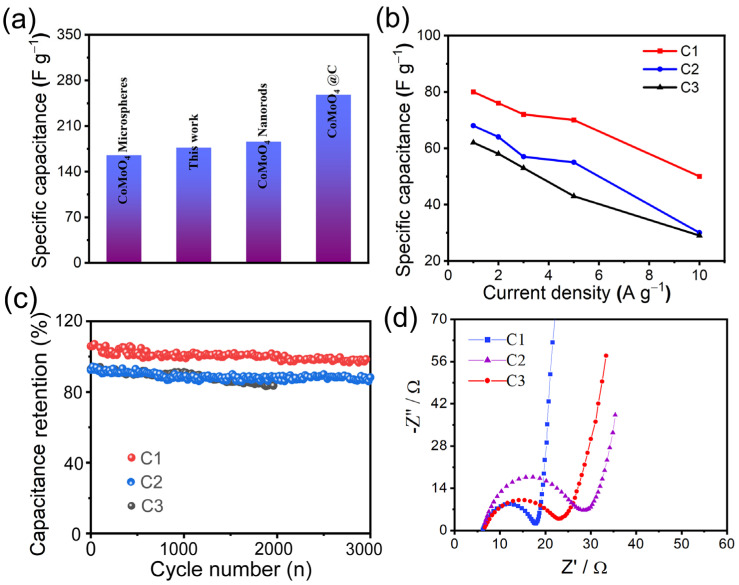
(**a**) A comparison of the recently reported electrode at a current density of 1 mA cm^−2^. (**b**) The rate performance. (**c**) The cycling performance of the electrodes at 1 A g^−1^. (**d**) Electrochemical impedance spectroscopy.

In order to research the electrochemical properties of the three samples, cyclic voltammetry (CV) and current-constant charge–discharge curve (GCD) tests were performed on electrode materials in a three-electrode system (Figure 4), and the rate performance and capacity of several electrode materials were evaluated according to the test results. As shown in Figure 4a–c, the CV curves concerning those three electrode materials at the scanning speed of 3, 5, 10, 20, and 30 mA cm^−2^ all show a pair of obvious redox peaks (Figure 4a–c), indicating that the electrode materials have redox behavior during their energy storage process. It is worth noting that C1 prepared with Co(NO_3_)_2_·6H_2_O as the raw material exhibited the largest CV area. Simultaneously, sample C1 presented an obvious negative peak shift, indicating that CoMoO_4_ prepared with Co(NO_3_)_2_·6H_2_O as the raw material has better electrochemical activity and faster reaction kinetics. Figure 4d−f shows the GCD curves concerning the above three electrode materials at the scanning speed of 3, 5, 10, 20, and 30 mV cm^−2^. According to Figure 4d,e, the GCD curves concerning all electrode materials presented good symmetry and obvious charging and discharging platforms, indicating that the three electrode materials have pseudocapacitance behaviors in their electrochemical reaction process and, therefore, belong to pseudocapacitance materials. They feature excellent energy storage characteristics and good charging and discharging characteristics and keep consistent with the CV test results. Moreover, the discharge duration of sample C1 is far longer than that of other electrode materials. When the current density was 1 A g ^−1^, the specific capacitance was 177 F g^−1^, which was higher than that of samples C2 (165 F g^−1^) and C3 (158 F g^−1^), respectively. The sample prepared by this method has almost no significant difference from similar electrodes reported in the recent literature (Figure 5a). At the same time, sample C1 also has good magnification performance and cycle stability (Figure 5b,c). This is possibly because nitrate was slower during the reaction, resulting in a more uniform rod-like structure in the sample. Furthermore, a great quantity of resultant oxygen deficiency accelerates the electron transfer rate and realizes the optimal capacitive performance.

Additionally, the charge transfer kinetics of electrode materials during the electrochemical reaction was further explored by electrochemical impedance spectroscopy (EIS) on electrode material in a frequency range from 10^−2^ to 10^4^ Hz. As shown in Figure 5d, EIS was divided into two parts, namely, the high-frequency region (semicircle) and the low-frequency region (slash). The intercept on the X-axis was the internal resistance of the entire system (Rs), including the contact impedance, the electrolyte impedance, and the impedance between the electroactive material and substrate. The semicircle size is relevant to the charge transfer impedance (Rct) of the redox reaction on the interface of electrode material. In the low-frequency region, the slash slope was determined by the Warburg impedance (W) generated from ion diffusion in the electrolyte [45,46]. According to the test results, the charge transfer resistances (Rct) in the high-frequency region were 14.6 Ω sample C1), 18.8 Ω (sample C2), and 26.9 Ω (sample C3), respectively, proving that sample C1 presents a fast electron transfer capacity during the electrochemical process. This may be because C1 nanorods have better hydrophilicity and more defects, thereby accelerating the lattice oxygen migration and the ion conductivity transfer. As a result, it presents relatively good electrical conductivity, thereby keeping consistent with the test results from current-constant charge and discharge tests.

### 2.3. Research on Thermal Stability

**Figure 6:**
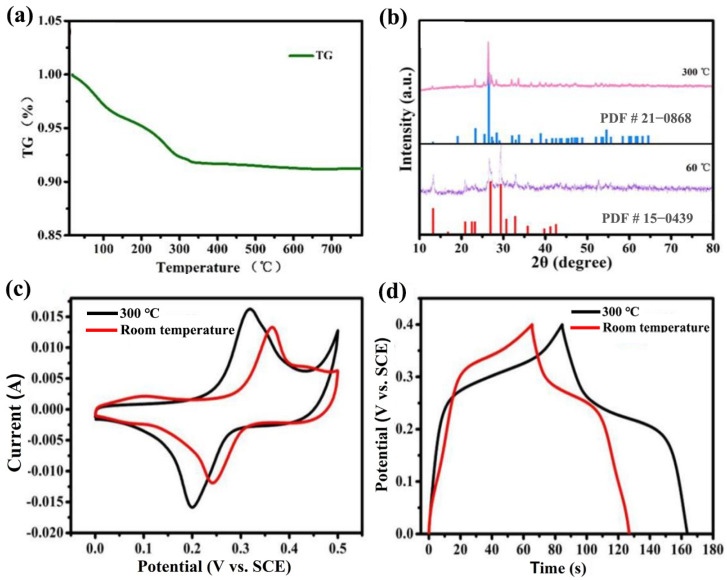
(**a**) Thermogravimetric curves, (**b**) XRD spectra, (**c**) CV curves, and (**d**) GCD curves of C1.

In order to examine the thermal stability of the rod-like CoMoO_4_ materials synthesized by the solid-phase chemical method at ambient temperature, a thermogravimetric test was conducted. According to Figure 6a, when the temperature rises from room temperature to 800 °C in an air atmosphere, the weight loss ratio before 150 °C is about 5%, which may be the loss of free water in the sample. When the temperature rises to 300 °C, the weight loss of the sample is complete, and the weight loss is 4% between 150 °C and 300 °C. This may be due to the residue on the surface of the sample, which is transformed into gas and separated from the sample in the process of rising temperature, resulting in its weight loss. Moreover, it can be seen in Figure 6a that the sample basically tended to stabilize after 300 °C. For this reason, this temperature was taken as its calcination temperature for research. The samples before and after calcination were tested by XRD. According to the test results, after calcination at 300 °C, strong diffraction peaks appeared at 23.1° and 26.3° (Figure 6b), consistent with the standard cards of CoMoO_4_ (PDF#21−0868). Simultaneously, the diffraction peak intensity of calcined samples is obviously enhanced, and the crystal pattern changes obviously. These results prove that the sample obtained by the solid-phase reaction at ambient temperature contains a lot of crystal water with unstable structure, and water loss and phase transformation can occur as the sample is subject to high temperature.

According to the electrochemical test in Figure 6c, the redox peak area of the sample after calcination at 300 °C was significantly larger than that before calcination at 1 mA cm^−2^ with a low current density. Meanwhile, according to the current-constant charge and discharge curves in Figure 6d, the discharge duration of sample C1 after calcination at 300 °C was longer than that before calcination. As a result, the thermal treatment exerts a great influence on the electrochemical properties of the CoMoO_4_ sample because the calcination process not only removes the free water inside the CoMoO_4_ sample but also improves its electrical conductivity. Simultaneously, some impurities inside the sample decompose and form a porous structure, which provides more channels for both electrolyte adsorption and ion transport, increasing the specific surface area of the electrode material and thereby resulting in the enhancement in specific capacitance.

## 3. Materials and Methods

### 3.1. Chemicals

Cobalt nitrate (Co(NO_3_)_2_·6H_2_O), sodium molybdate (Na_2_MoO_4_·2H_2_O), cobalt sulfate (CoSO_4_·7H_2_O), cobalt chloride (CoCl_2_·6H_2_O), potassium hydroxide (KOH), and absolute ethyl alcohol (EtOH) were purchased from Aladdin Ltd. of Shanghai in China. All chemicals were used as received without further purification. In all experiments, deionized water was used from a millipore.

### 3.2. Synthesis of CoMoO_4_ Nanomaterial

Firstly, 10 mmol of Co(NO_3_)_2_·6H_2_O (2.9103 g) and Na_2_MoO_4_·2H_2_O (2.4195 g) were accurately weighed and ground in an agate mortar for 1 h. During the solid-phase reaction process, with the progress in this chemical reaction, the color and state of the reaction system gradually turned from pink solid powder into purple solid powder. In order to realize a full reaction of those raw materials, the ground product was sealed in a conical bottle for a hot water bath at 60 °C for 24 h. Finally, the solid powder product after full reaction was rinsed with adequate deionized water, dried at the ambient temperature, and then named C1 for further utilization. For comparison, the 10 mmol Co(NO_3_)_2_·6H_2_O was replaced by 10 mmol of CoSO_4_·7H_2_O (2.811 g) and CoCl_2_·6H_2_O (2.3793 g) via a similar method, and the obtained samples were named C2 and C3, respectively.

### 3.3. Electrode Preparation and Electrochemical Test

#### 3.3.1. Electrode Preparation

When the working electrode of the Faraday pseudocapacitor was prepared through the coating method, the commercially available nickel mesh was taken as the base, which was cut as per the size of 1 × 3 cm before use. Then, the nickel mesh was ultrasonically cleaned in hydrochloric acid, acetone, ethanol, and deionized water for 8 h to remove surface oxides and dried at 60 °C. The prepared electrode material, acetylene black, and adhesive (PTFE) were accurately weighed based on the mass ratio of 8:1:1 and then placed in an agate mortar, which, after adding a certain amount of anhydrous ethanol, was mixed and ground for 0.5 h to make the substances evenly mixed. As the active substance mixture took the shape of a thick paste, it was evenly coated on the treated nickel mesh with a coating area of 1 × 1 cm^2^. Finally, the coated nickel mesh was rolled over by a tablet press at a pressure of 20 MPa to obtain the working electrode.

#### 3.3.2. Electrochemical Test

A standard three-electrode system was adopted to test the electrochemical performance. A platinum electrode was adopted as the counter electrode and a calomel electrode was adopted as the reference electrode. Prepared nickel mesh was adopted as the working electrode. The test was conducted in a 2 M KOH electrolyte via an electrochemical workstation model CHI-760D with a CV test voltage range from 0 to 0.5 V.

### 3.4. Sample Characterization

#### 3.4.1. Phase Composition and Structure Analysis

The phase, structure, and composition of the sample were analyzed by an X-ray diffraction (XRD) analyzer produced by Bruker from Germany. The sweep range of the XRD test was 2θ = 10–80°, and the step size and sweep time were adjusted according to the signal and noise conditions of the test sample. The microstructure and morphology of electric samples were characterized by FE-SEM produced by a Japanese electronics company.

#### 3.4.2. Analyses of Element Valence, Content, and Deficiency

The binding energy differences in different valence states of different elements were determined by X-ray photoelectron spectroscopy (XPS) to identify the chemical bond and electron transfer of samples. Then, according to the differences in peak strengths at different binding energies, the relative contents of different chemical bonds in those samples were determined. The instrument model adopted herein was ESCALLAB 250xi produced by Thermo Fisher Company in the Waltham, MA, USA. The binding energy of all elements obtained was calibrated with C 1s (284.8 eV) as the carbon standard. Thermo Avantage software (59918) was adopted to perform peak sub-fitting on the original spectra.

## 4. Conclusions

In this paper, three different kinds of CoMoO_4_ nanorods were prepared at a low temperature via simple and environmentally friendly solid-phase chemical reactions with solid inorganic salts as raw materials. Structural characterization and electrochemical property tests show that the samples synthesized with Co(NO_3_)_2_·6H_2_O as the raw material presented a relatively large specific capacitance (177 F g^−1^). This may be because the reaction rate of nitrate was slow during the reaction process, and its crystal had difficulties in agglomerating when growing. Thus, the prepared CoMoO_4_ nanomaterials had a more uniform structure, and its structure was too tough to collapse during both the charge and discharge processes, so its capacitor presented optimal performance. This method can lay a theoretical foundation for the green synthesis of other two-element transitional metal oxide electrode materials and thereby provide a new train of thought.

## Data Availability

Data are contained within this article.
